# METTL7B is a novel prognostic biomarker of lower-grade glioma based on pan-cancer analysis

**DOI:** 10.1186/s12935-021-02087-4

**Published:** 2021-07-19

**Authors:** Zhipeng Jiang, Wen Yin, Hecheng Zhu, Jun Tan, Youwei Guo, Zhaoqi Xin, Quanwei Zhou, Yudong Cao, Zhaoping Wu, Yirui Kuang, Can Li, Dongcheng Xie, Hailong Huang, Ming Zhao, Xingjun Jiang, Lei Wang, Caiping Ren

**Affiliations:** 1 Department of Neurosurgery, Xiangya Hospital of Central South University, Changsha, 410008 Hunan China; 2 Changsha Kexin Cancer Hospital, Changsha, 410205 Hunan China; 3 Cancer Research Institute, Collaborative Innovation Center for Cancer Medicine, The Key Laboratory for Carcinogenesis of Chinese Ministry of Health and the Key Laboratory of Carcinogenesis and Cancer Invasion of the Chinese Ministry of Education, School of Basic Medical Science, Central South University, Changsha, Hunan People’s Republic of China

**Keywords:** Glioma, Methyltransferase-like 7B, Pan-cancer analysis, Prognostic markers, Epithelial–mesenchymal transition (EMT), Immune cell infiltration

## Abstract

Methyltransferase-like 7B (METTL7B) is a member of the methyltransferase-like protein family that plays an important role in the development and progression of tumors. However, its prognostic value and the correlation of METTL7B expression and tumor immunity in some cancers remain unclear. By analyzing online data, we found that METTL7B is abnormally overexpressed in multiple human tumors and plays an important role in the overall survival (OS) of patients with 8 cancer types and disease-free survival (DFS) of patients with 5 cancer types. Remarkably, METTL7B expression was positively correlated with the OS and DFS of patients with lower-grade glioma (LGG). In addition, a positive correlation between METTL7B expression and immune cell infiltration in LGG was observed. Moreover, we identified a strong correlation between METTL7B expression and immune checkpoint gene expression in kidney chromophobe (KICH), LGG and pheochromocytoma and paraganglioma (PCPG). Furthermore, METTL7B was involved in the extracellular matrix (ECM) and immune-related pathways in LGGs. Finally, in vitro experiments showed that knockdown of METTL7B inhibited the growth, migration, invasion and the epithelial–mesenchymal transition (EMT) of LGG cells. METTL7B expression potentially represents a novel prognostic biomarker due to its significant association with immune cell infiltration in LGG.

## Introduction

methyltransferase-like 7B (METTL7B) is a member of the methyltransferase-like protein family, which is considered related to the methylation of Golgi proteins and lipid metabolism. METTL7B is involved in infections, hepatitis and obstetric diseases [1–3]. METTL7B has also been proven to be overexpressed in multiple malignancies [e.g., papillary thyroid cancer (PTC), lung cancer, and esophageal adenocarcinoma], while it is downregulated in breast cancer [4–7]. Through different mechanisms, METTL7B plays a vital regulatory role in multiple tumors. Ye et al. found that METTL7B may promote tumor invasion and malignancy by activating the TGF-β1-induced EMT in PTC [4]. METTL7B promotes tumorigenesis by regulating cell cycle progression in non-small cell lung cancer [8]. In a subsequent exploration of the relationship between METTL7B and lung adenocarcinoma (LUAD), Ali et al. reported that abnormal expression of the METTL7B gene obviously affects the proliferation, invasion and migration of tumor cells [5]. METTL7B is required for maintaining of the morphological integrity of the Golgi complex, a process that is regulated by changes in the RhoBTB1 level. McKinnon reported that a lack of METTL7B expression is associated with Golgi apparatus fragmentation and breast cancer cell invasion [6]. Although studies have been conducted on PTC, breast cancer and lung cancer, the role of METTL7B in other cancers is still poorly understood.

The tumor microenvironment (TME) is a sophisticated organizational structure that refers to the intracellular milieu produced by tumor cells and required for tumor cells to survive, including fibroblasts, tumor cells, inflammatory and immune cells and other types of cells around them, as well as intercellular stroma, extracellular matrix (ECM), microvessels and signaling biomolecules in nearby areas [9]. Tumor development and progression are affected by changes in the TME, which is produced by the dynamic interaction between malignant cells and normal cells. Tumor immunity, an important part of tumor therapy, is the study of specific immune cells, immune proteins and related signaling molecules in the tumor microenvironment. A large number of studies have shown that the efficacy of immunotherapy usually depends on the interaction between tumor cells and immune regulation within the tumor microenvironment [10, 11]. In chronic HIV infection, METTL7B is involved in the epigenetic modification of FOXP3, which induces immune regulation disorders [1]. Therefore, the underlying mechanism of METTL7B in tumor immunology must be discovered. Moreover, the EMT has been shown to promote cell migration and invasiveness and influences the TME by promoting the secretion of many factors [12]. Although reports have documented that the expression of METTL7B modulates tumor progression by inducing the EMT, the relationship between the METTL7B gene and tumor immunity requires further exploration [4].

The tumor tissue mutation burden (TMB) is calculated as a ratio of the number of gene mutations to the total length of exons, thus reflecting the number of mutations in a particular cancer genome. The TMB has been identified as a validated biomarker for predicting the efficacy of checkpoint inhibitors [13]. Microsatellite instability (MSI) is a genetic instability characterized by changes in the length of short nucleotide repeats (microsatellites) due to aberrant DNA mismatch repair. According to recent studies, patients with cancer presenting a high degree of MSI are more likely to experience a long-term survival benefit from immunotherapy. Thus, it has been considered an important indicator for the diagnosis, prognosis judgment and treatment selection of various tumors [14]. The association between the METTL7B gene and TMB and MSI requires further investigation.

**Fig. 1:**
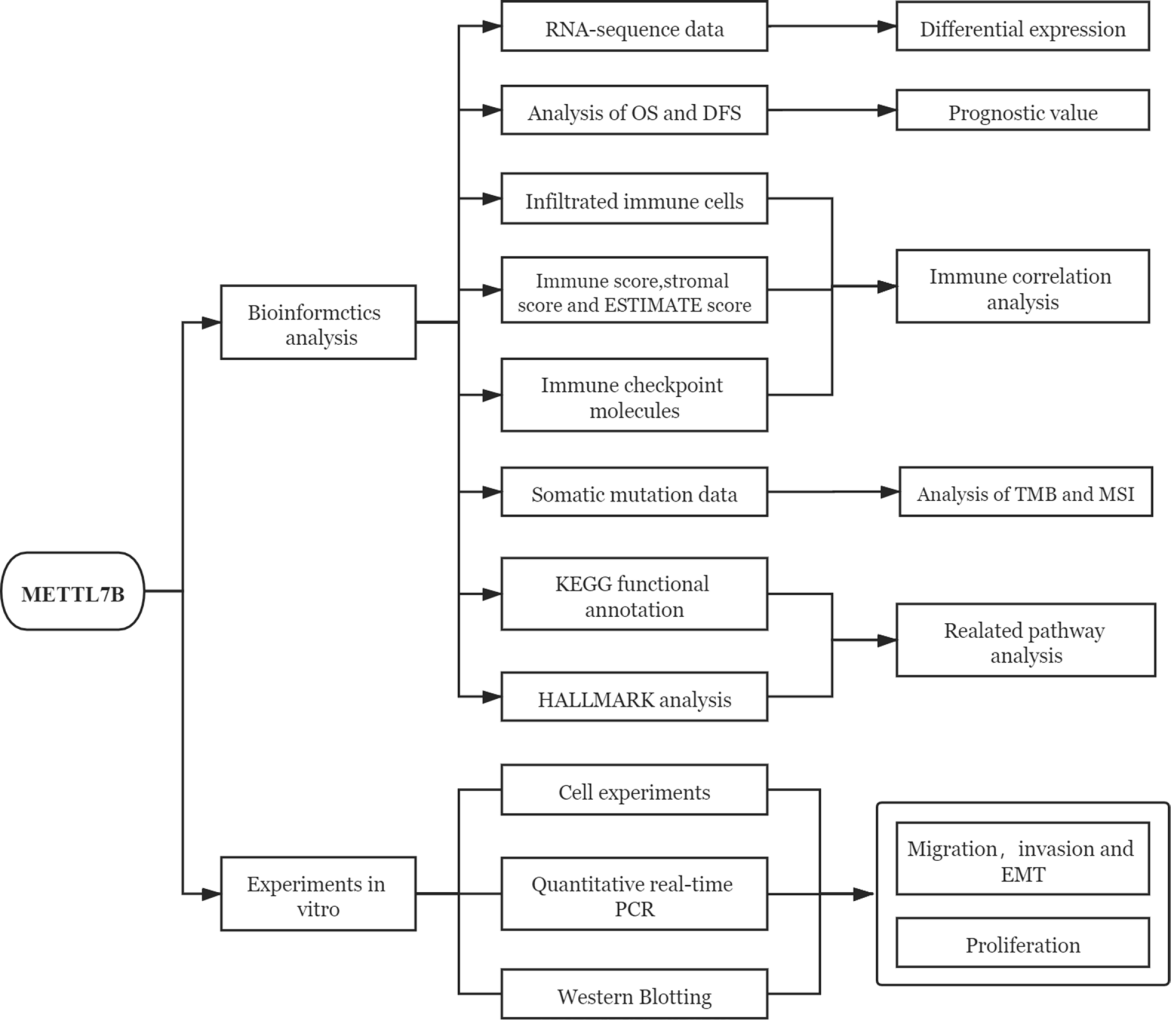
Flow chart of the research methodology and design

In the present study, we comprehensively analyzed METTL7B expression across 33 cancer types. Then, we used the Gene Expression Profiling Interactive Analysis (GEPIA) database to further explore the relationship between METTL7B expression and the cancer prognosis. Moreover, we further investigated the correlation between METTL7B expression and the levels of immune-related cells and cytokines in different cancers. The relationships between the expression of this gene and TMB and MSI were also evaluated. More importantly, we performed experimental validation and found that METTL7B contributed to cell proliferation, progression and EMT in lower-grade glioma (LGG). The findings of this study suggest that METTL7B is a potential prognostic biomarker and is closely associated with immune cell infiltration in various tumors, especially in LGG. A flow chart of the research methodology and design is shown in Fig. 1.

## Materials and methods

### Expression level analysis

The characteristics of the expression of the METTL7B gene in 31 normal tissues were obtained and analyzed by collecting the relevant data from the Genotype-Tissue Expression (GTEx) database (https://gtexport.org/home/). Similarly, the expression levels of the METTL7B gene in 21 tumor cell lines were collected and processed by consulting the Cancer Cell Line Encyclopedia (CCLE) database (https://portals.broadinstitute.org/ccle/about). An analysis of differentially expressed genes (DEGs) was performed using the data originating from the GTEx database and The Cancer Genome Atlas (TCGA) to identify the differential expression of METTL7B in normal and neoplastic tissue. The gene sequencing datasets, clinical data and information from follow-up for patients with 33 types of cancer were available in TCGA databases. (adrenocortical carcinoma: ACC; bladder urothelial carcinoma: BLCA; breast invasive carcinoma: BRCA; cervical squamous cell carcinoma: CESC; cholangiocarcinoma: CHOL; colon adenocarcinoma: COAD; lymphoid neoplasm diffuse large B cell lymphoma: DLBC; esophageal carcinoma: ESCA; glioblastoma multiforme: GBM; brain lower grade glioma: LGG; head and neck squamous cell carcinoma: HNSC; kidney chromophobe: KICH; kidney renal clear cell carcinoma: KIRC; kidney renal papillary cell carcinoma: KIRP; acute myeloid leukemia: LAML; liver hepatocellular carcinoma: LIHC; lung adenocarcinoma: LUAD; lung squamous cell carcinoma: LUSC; mesothelioma: MESO; ovarian serous cystadenocarcinoma: OV; pancreatic adenocarcinoma: PAAD; pheochromocytoma and paraganglioma: PCPG; prostate adenocarcinoma: PRAD; rectum adenocarcinoma: READ; sarcoma: SARC; skin cutaneous melanoma: SKCM; stomach adenocarcinoma: STAD; testicular germ cell tumors: TGCT; thyroid carcinoma: THCA; thymoma: THYM; uterine corpus endometrial carcinoma: UCEC; uterine carcinosarcoma: UCS; and uveal melanoma: UVM).

### Evaluation of the prognostic value

We used the GEPIA database to collect data on overall survival (OS) and disease-free survival (DFS) for patients with 33 different types of tumors and different levels of METTL7B expression, and generated Kaplan–Meier plots to determine the effects of METTL7B expression on the OS and DFS of patients with these cancers.

### Immune correlation analysis

As a comprehensive resource to estimate immune cell abundance, the Tumor Immune Evaluation Resource (TIMER) database was used to analyze the relationship between different gene expressions levels and immune cell infiltration (https://cistrome.shinyapps.io/timer/) [15]. Similarly, we used the TIMER database to measure the level of infiltration of 6 major immune cell types in different tumor tissues, which included B cells, CD4+ T cells, CD8+ T cells, neutrophils, macrophages, and dendritic cells. Furthermore, we calculated the Spearman correlation coefficient to assess correlations between METTL7B expression levels and immune cell infiltration.

We used Estimation of STromal and Immune cells in MAlignant Tumors using Expression data (ESTIMATE) algorithm to estimate the proportions of infiltrating stromal and immune cells in the entire tumor microenvironment based on the gene expression profile [16]. The immune score, stromal score and ESTIMATE score were subjected to a Spearman correlation analysis. Moreover, we also conducted a Pearson correlation analysis to detect the association between METTL7B expression and immune checkpoint expression in the 33 types of cancer.

### Analysis of correlations with the tumor mutation burden (TMB) and microsatellite instability (MSI)

The TMB was calculated as the number of mutations divided by the total length of the exons (corrected to the number of mutated bases per 1 million bases). We analyzed the relationship between METTL7B expression and the TMB of all TCGA patients. Furthermore, we obtained the MSI score from TCGA. The results of the analysis of TMB and MSI were visualized in a radar map.

### Gene set enrichment analysis (GSEA) in LGG

An enrichment analysis is a series of research methods that relate a group of genes to a functional description. The Gene set enrichment analysis (GSEA) website (https://www.gsea-msigdb.org/gsea/downloads.jsp) is a public data analysis site, where we downloaded the Kyoto Encyclopedia of Genes and Genomes (KEGG) and HALLMARK gene sets. A GSEA was conducted on the LGG dataset to investigate the potential KEGG pathways and HALLMARK terms between groups with high and low METTL7B expression. Only gene sets with a NOM *p* < 0.05, FDR *q* < 0.25, and |NES|> 1 were considered to be statistically significant.

### Cell culture and quantitative real-time PCR

Glioma cell lines (HS683 and SHG44) were provided by Xiangya Medical School of Central South University, Changsha, China. HS683 and SHG44 cells were cultured in high-glucose DEME (Gibco) containing 10% fetal bovine serum. The siRNAs against the METTL7B gene were purchased from RiboBio Corporation (Guangzhou, China). Lipofectamine 2000 transfection reagent (Thermo Fisher Scientific) was used for the siRNA transfection. We used the TRIzol lysis method to extract total RNA from cells, and total RNA was reverse transcribed to cDNAs using the Thermo Scientific RevertAid First Strand cDNA Synthesis Kit (Thermo Scientific, Waltham, MA). Quantitative real-time PCR (qRT-PCR) was used to detect levels of the METTL7B mRNA according to the manufacturer’s protocol (SYBR Green Master Mix, Vazyme). Gene expression levels were calculated using the 2-ΔΔCt method. The primers were purchased from Sangon (Shanghai, China), and the sequences used for qPCR were as follows: for METTL7B, the forward primer was 5ʹ-CCTGCCTAGACCCAAATCCC-3ʹ and the reverse primer was 5ʹ-AAACCGCTCATATTGGAGGTG-3ʹ for GAPDH, the forward primer was 5ʹ-CATTGACCTCAACTACATGGTT-3ʹ and the reverse primer was 5ʹ-CCATTGATGACAAGCTTCCC-3ʹ.

### Wound healing and Transwell assays

We conducted wound healing and Transwell assays using previously described methods [17, 18].

### Western blotting

Western blot assays were performed as described previously [19]. Antibodies against GAPDH, Vimentin, and N-cadherin were purchased from ProteinTech Group (Chicago, IL, USA).

### Cell colony formation assay

The independent growth ability of cells was determined by performing colony formation experiments. Cells transfected with METTL7B or control siRNAs, (1000 cells per well) were plated into 6-well plates. The medium was replaced as needed. After 10–14 days of culture, the cell colonies were fixed with 4% paraformaldehyde. Then, colonies were stained with 0.01% crystal violet and counted to judge the cell growth ability.

### Cell proliferation assay

The Cell Counting Kit-8 (CCK-8) assay was conducted to determine the cell proliferation. HS683 and SHG44 cells were transfected with METTL7B or control siRNAs. Twenty-four hours after transfection, HS683 and SHG44 cells (2000 cells/well) were seeded into 96-well plates. Then, 10 μL of CCK-8 regent were added to each well. After incubation for 2 h at 37 °C, the absorbance was measured at a wavelength of 450 nm. The assay was repeated every 24 h.

### Statistical analysis

The expression of METTL7b in different normal tissues and neoplastic cell lines was evaluated using the Kruskal–Wallis test. The difference in the expression of METTL7B in normal and neoplastic tissues was compared using a t-test and normalized by log2 transformation. The prognostic value of the METTL7B gene was analyzed by performing a univariate survival analysis. Pearson’s and Spearman’s correlation coefficients were calculated to analyze correlations. In all data analyses, *p* < 0.05 was considered statistically significant.

## Results

### METTL7B expression in multiple human cancers

**Fig. 2:**
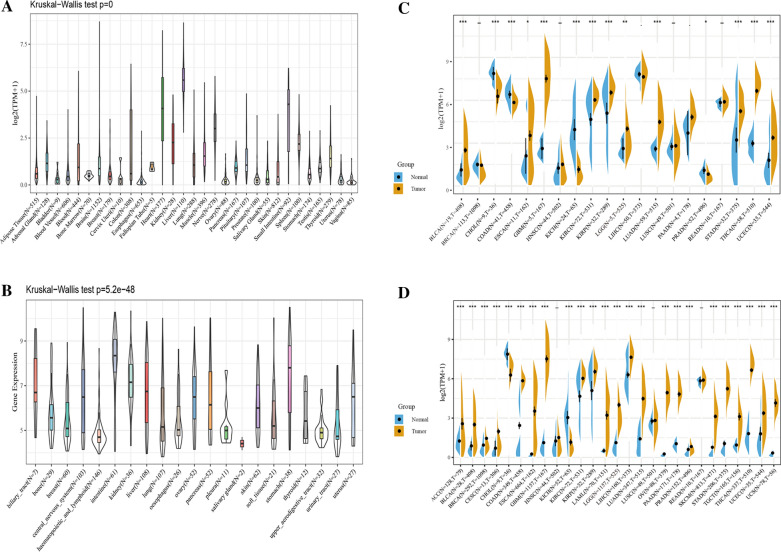
The expression levels of METTL7B in the human pan-cancer dataset. **A** METTL7B expression in 31 normal human tissues in the GTEx data set. **B** METTL7B expression in 21 tumor cells in the CCLE dataset. **C** Differential expression of METTL7B in normal tissues and cancers in TCGA datasets. **D** METTL7B was significantly upregulated in 22 cancer types from the TCGA and GTEx databases (*P < 0.05, **P < 0.01, and ***P < 0.001)

First, we employed the GTEx database to investigate the expression levels of METTL7B in 31 types of tissues. Our results showed a relative overexpression of METTL7B in several normal tissues, including heart, liver, nerve and small intestine (Fig. 2A). Next, we detected the expression of METTL7B in 21 neoplastic cells using the CCLE database. As shown in Fig. 2B, all 21 neoplastic cell lines expressed the METTL7B gene. In an effort to confirm the differential expression of the METTL7B gene in tumor and normal tissues, we analyzed the METTL7B expression level based on TCGA data. Figure 2C shows an evident increase in METTL7B expression in BLCA, GBM, LUAD, STAD, THCA, and UECE compared with normal tissues. In contrast, it was expressed at much lower levels in CHOL, COAD and KICH than in normal tissues. We conducted an integrated analysis of 27 groups of tissue data from the GTEx and TCGA databases to further explore the difference in METTL7B expression in a relatively large set of tissues samples. The results indicated upregulated expression of METTL7B in 22 tumors (ACC, BLCA, BRCA, CESC, COAD, ESCA, GBM, KIRC, KIRP, LAML, LGG, LIHC, LUAD, OV, PAAD, PRAD, SKCM, STAD, TGCT, THCA, UCEC and UCS) compared with normal tissues (Fig. 2D). Collectively, METTL7B is aberrantly overexpressed in most cancer types.

### Prognostic value of METTL7B in multiple human cancers

**Fig. 3:**
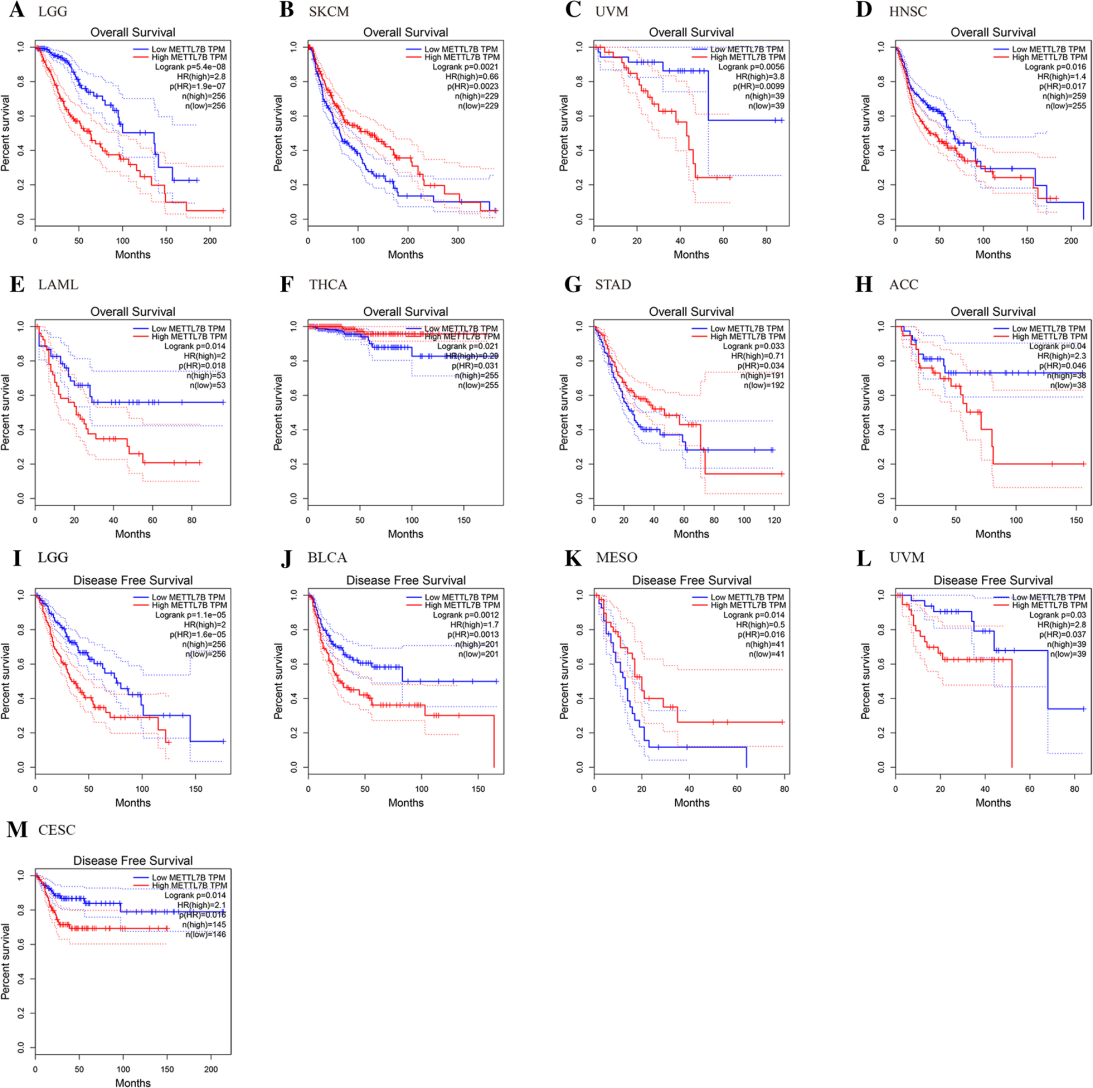
Survival analyses based on differences in METTL7B expression in patients with different cancers. **A**–**H** OS, overall survival; **I**–**M** DFS, disease-free survival

We compared OS and DFS between patients with different METTL7B expression levels to evaluate the prognostic value of METTL7B expression levels across cancers. Notably, higher expression of METTL7B was an unfavorable indicator for the OS of patients with LGG (OS: log-rank p = 5.4e−08, HR = 2.8), UVM (OS: log-rank p = 0.0056, HR = 3.8), HNSC (OS: log-rank p = 0.016, HR = 1.4), LAML (OS: log-rank p = 0.014, HR = 2) and ACC (OS: log-rank p = 0.04, HR = 2.3), but the opposite was true in patients with SKCM (OS: log-rank p = 0.0021, HR = 0.66), THCA (OS: log-rank p = 0.021, HR = 0.29) and STAD (OS: log-rank p = 0.033, HR = 0.71) (Fig. 3A–H). Moreover, higher expression of METTL7B was a marker for shorter DFS in patients with LGG (DFS: log-rank p = 1.1e−05, HR = 2), BLCA (DFS: log-rank p = 0.0012, HR = 1.7), UVM (DFS: log-rank p = 0.03, HR = 2.8) and CESC (DFS: log-rank p = 0.014, HR = 2.1), but the opposite was true in patients with MESO (DFS: log-rank p = 0.014, HR = 0.5). Therefore, the METTL7B gene may be closely related to the prognosis and progression of LGG.

### Correlation between METTL7B expression and immune cell infiltration and immune checkpoint marker expression

**Fig. 4:**
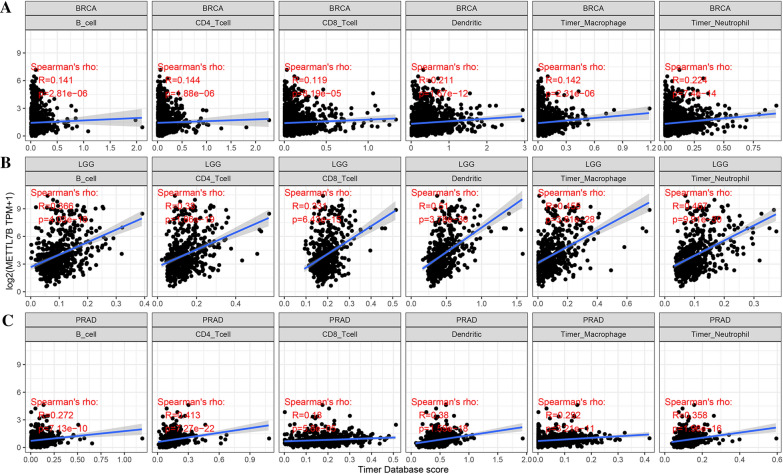
METTL7B expression is positively correlated with immune cell infiltration in cancers. **A** BRCA. **B** LGG. **C** PRAD

**Fig. 5:**
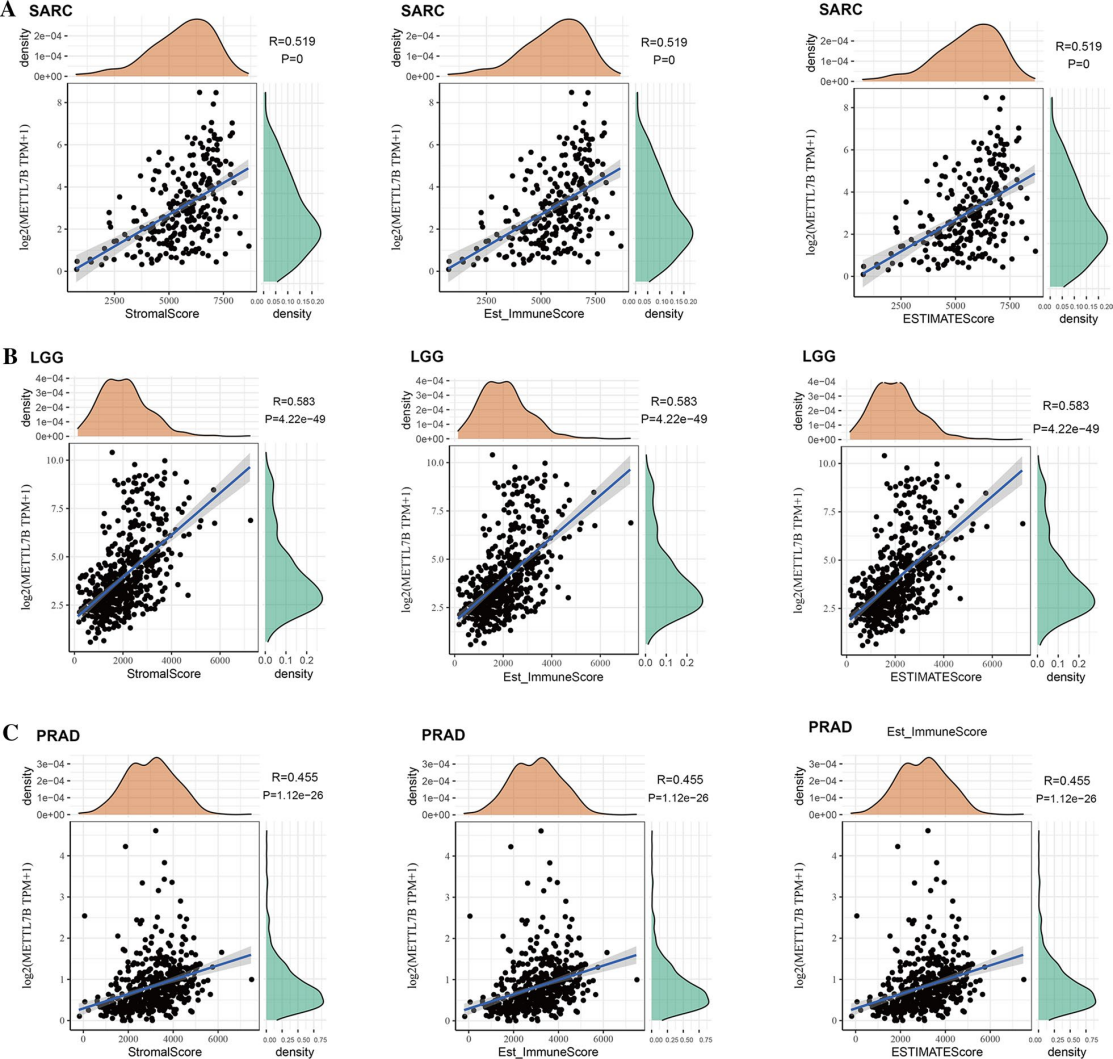
Correlations of the immune score, stromal score, and ESTIMATE scores with METTL7B expression in the top three cancers. **A** SARC. **B** LGG. **C** PARD

We analyzed the related scores of 6 immune cell types across cancers using the online TIMER database to determine the correlation between METTL7B expression and immune cell infiltration level in diverse tumor types. As presented in Fig. 4, the METTL7B expression levels and levels of infiltrating immune cells were positively correlated in multiple cancers (top three cancers: BRCA, LGG and PRAD). In particular, in LGG, the expression of METTL7B was significantly related to the levels of infiltrating B cells (R = 0.366, p = 4.03e−18), CD4+ T cells (R = 0.38, p = 1.86e−19), CD8+ T cells (R = 0.331, p = 6.43e−15), dendritic cells (R = 0.51, p = 3.78e−36), macrophages (R = 0.455, p = 3.81e−28), and neutrophils (R = 0.467, p = 9.91e−30). We further analyzed three prognostic factors (the stromal score, immune score and ESTIMATE score) in these cancers. Our results illustrate that a greater number of infiltrated immune cells was correlated with high METTL7B expression (Fig. 5).

**Fig. 6:**
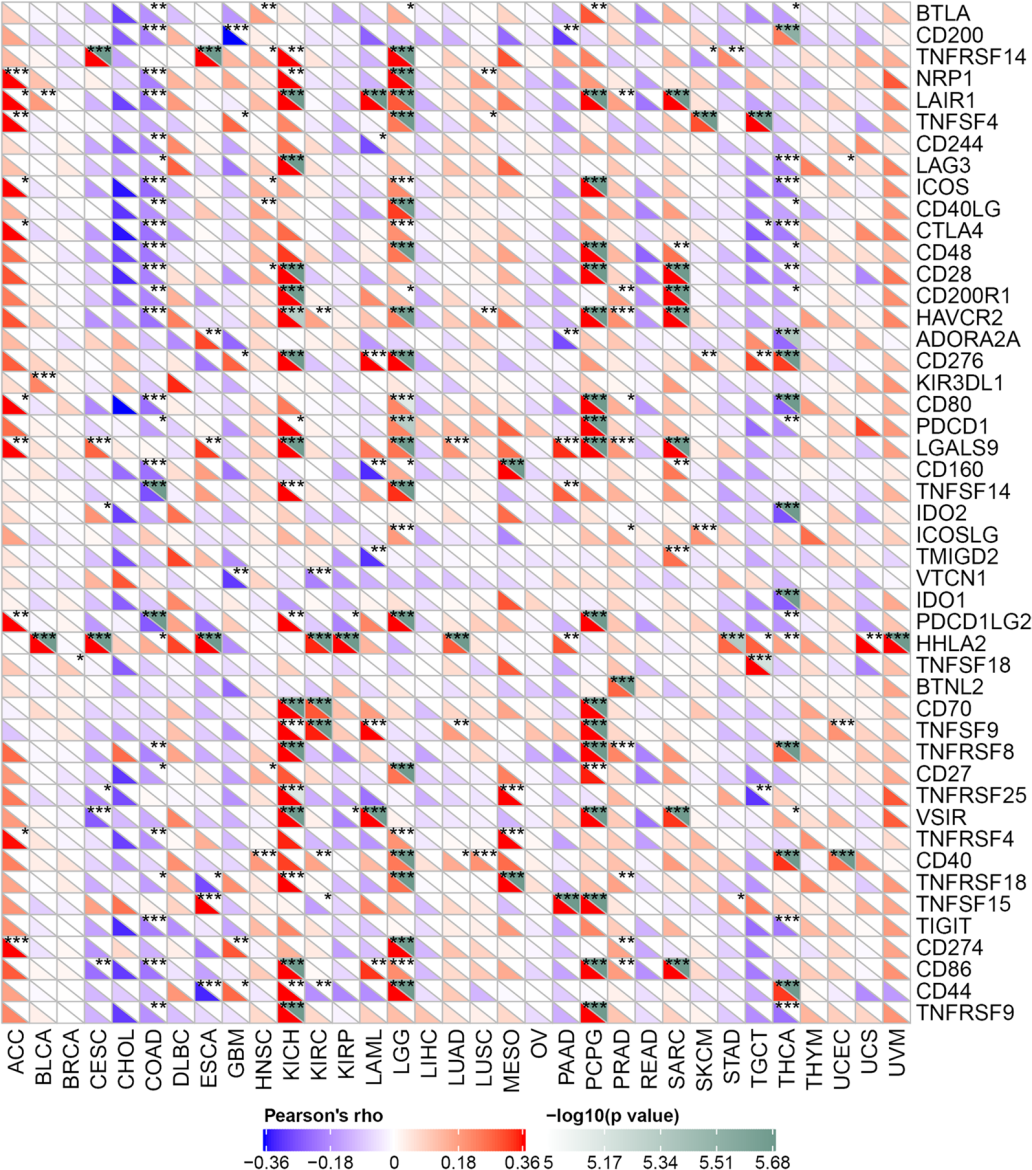
Correlation between METTL7B expression and immune checkpoint molecule expression

Immune checkpoint molecules maintain the activation of the immune system within the normal range. The abnormal expression of immune checkpoint molecules is closely related to the occurrence and development of some tumors, representing a potential treatment option for patients with various types of malignancies. Therefore, we conducted a correlation analysis between METTL7B expression and immune checkpoint molecule expression in 33 cancer types. In particular, the expression of METLL7B was positively correlated with the expression of a plethora of immune checkpoint molecules, such as LGALS9, CD70, CD27 and CD86, in KICH, LGG and PCPG. In contrast, in COAD and THCA, METLL7B expression was negatively correlated with a series of immune checkpoint molecules, such as CTLA4, CD8 and TIGIT (Fig. 6). In summary, the METTL7B gene is very important in regulating tumor immunity, which might explain its influence on the prognosis and survival of patients.

### Correlation between METTL7B expression and TMB and MSI

**Fig. 7:**
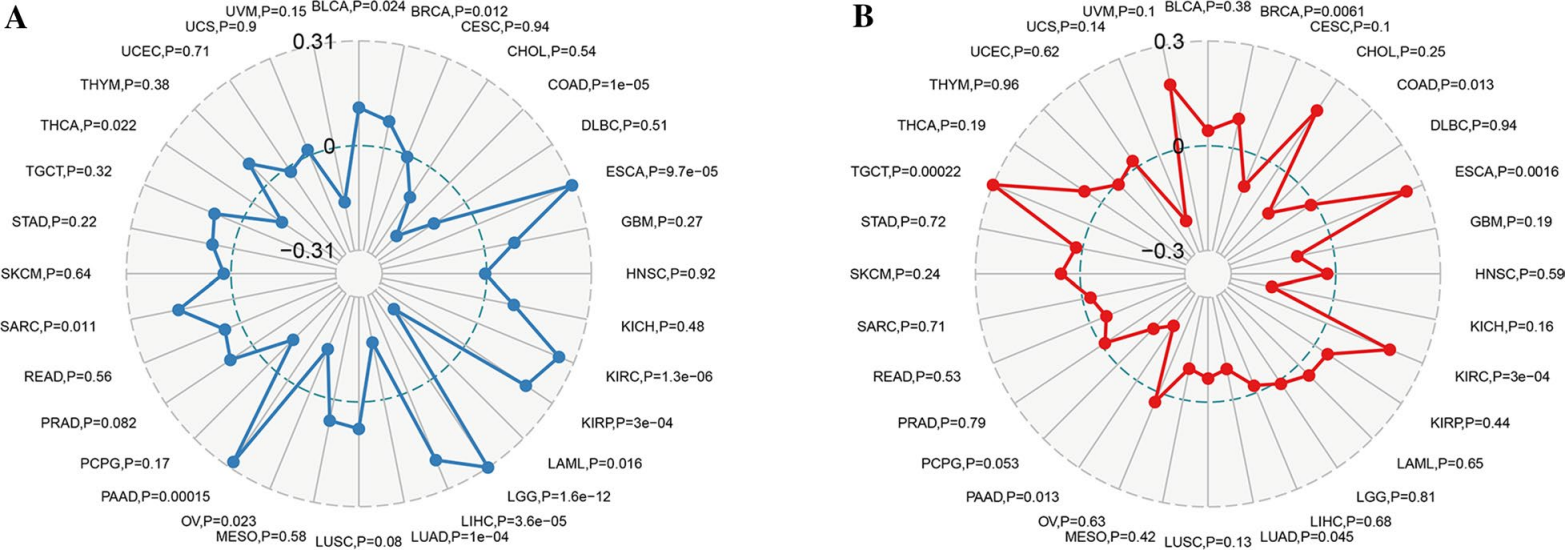
Correlations between METTL7B expression and the TMB and MSI in various cancers. **A** Radar map of the correlation between METTL7B expression and the TMB. **B** Radar map of the correlation between METTL7B expression and MSI

The tumor tissue mutational burden (TMB) is also a potential biomarker of ICIs in many neoplasms and plays a promising role in tumor immunotherapy. Hence, further studies are needed to examine the correlation between METTL7B expression levels and the TMB. As presented in Fig. 7A, a significant positive correlation existed between METTL7B expression and the TMB in BLCA, BRCA, ESCA, KIRC, KIRP, LGG, LIHC, PAAD and SARC. Conversely, the expression level of METTL7B showed a negative correlation with the TMB in 4 types of neoplasms, including COAD, LAML, LUAD, and THCA. In addition, MSI, an indicator of genetic instability, is also helpful to screen patients for immunotherapy. As shown in Fig. 7B, the METTL7B expression level was significantly positively correlated with MSI in BRCA, ESCA, KIRP and TGCT, but negatively correlated with MSI in COAD, LUAD, PAAD, and PCPG. Our results revealed that METTL7B might have a certain correlation with the TMB in LGG. However, the data for the correlation with the MSI in LGG were insensitive and therefore did not detect an association with METTL7B.

### Analysis of pathways related to METTL7B in LGG

**Fig. 8:**
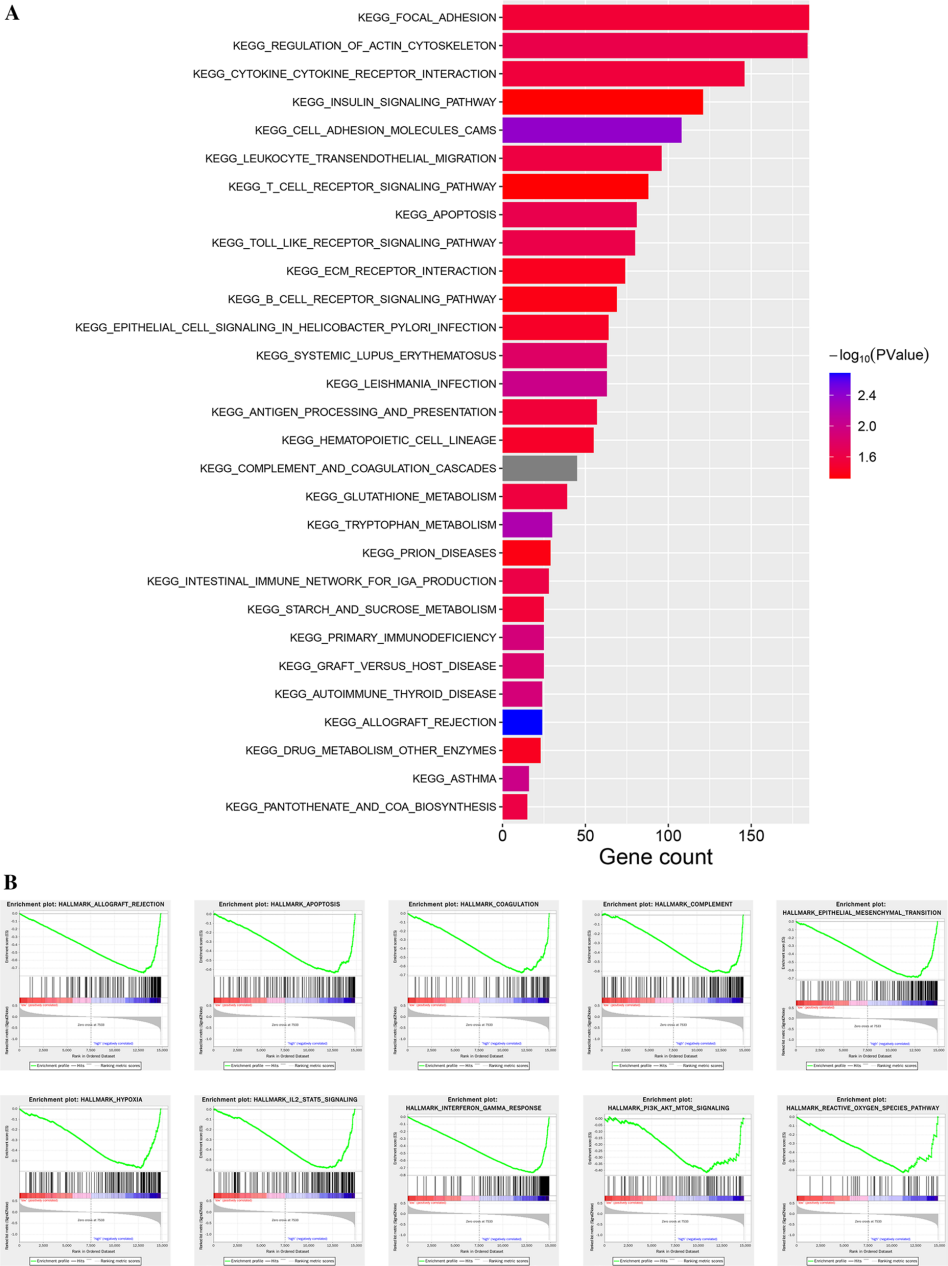
GSEA for comparing KEGG pathways and HALLMARK terms between the low and high METTL7B groups. **A** Twenty-nine KEGG pathways were significantly enriched in the high METTL7B group. **B** The top 10 enriched HALLMARK terms in the high METTL7B group

We performed a GSEA to identify the functional enrichment of pathways related to high METTL7B expression and low METTL7B expression based on the expression profile of LGG. In the context of the KEGG functional annotation, 29 pathways were significantly enriched in the high-expression group (Fig. 8A). The following top 5 enriched pathways were identified: FOCAL ADHESION, REGULATION OF ACTIN CYTOSKELETON, CYTOKINE-CYTOKINE RECEPTOR INTERACTION, INSULIN SIGNALING PATHWAY, and CELL ADHESION MOLECULES CAMS. Based on the results of HALLMARK analysis, ALLOGRAFT REJECTION, APOPTOSIS, COAGULATION, COMPLEMENT, EPITHELIAL–MESENHCAYMAL TRANSITION, HYPOXIA, IL2/STAT5 SIGNALING, INTERFERON GAMMA RESPONSE, PI3K/AKT/MTOR SIGNALING, and REACTIVE OXYGEN SPECIES PATHWAY were the top 10 enriched terms (Fig. 8B). However, no KEGG pathways or HALLMARK terms were enriched in the low expression group.

### METTL7B promotes LGG cell migration, invasion and EMT

**Fig. 9:**
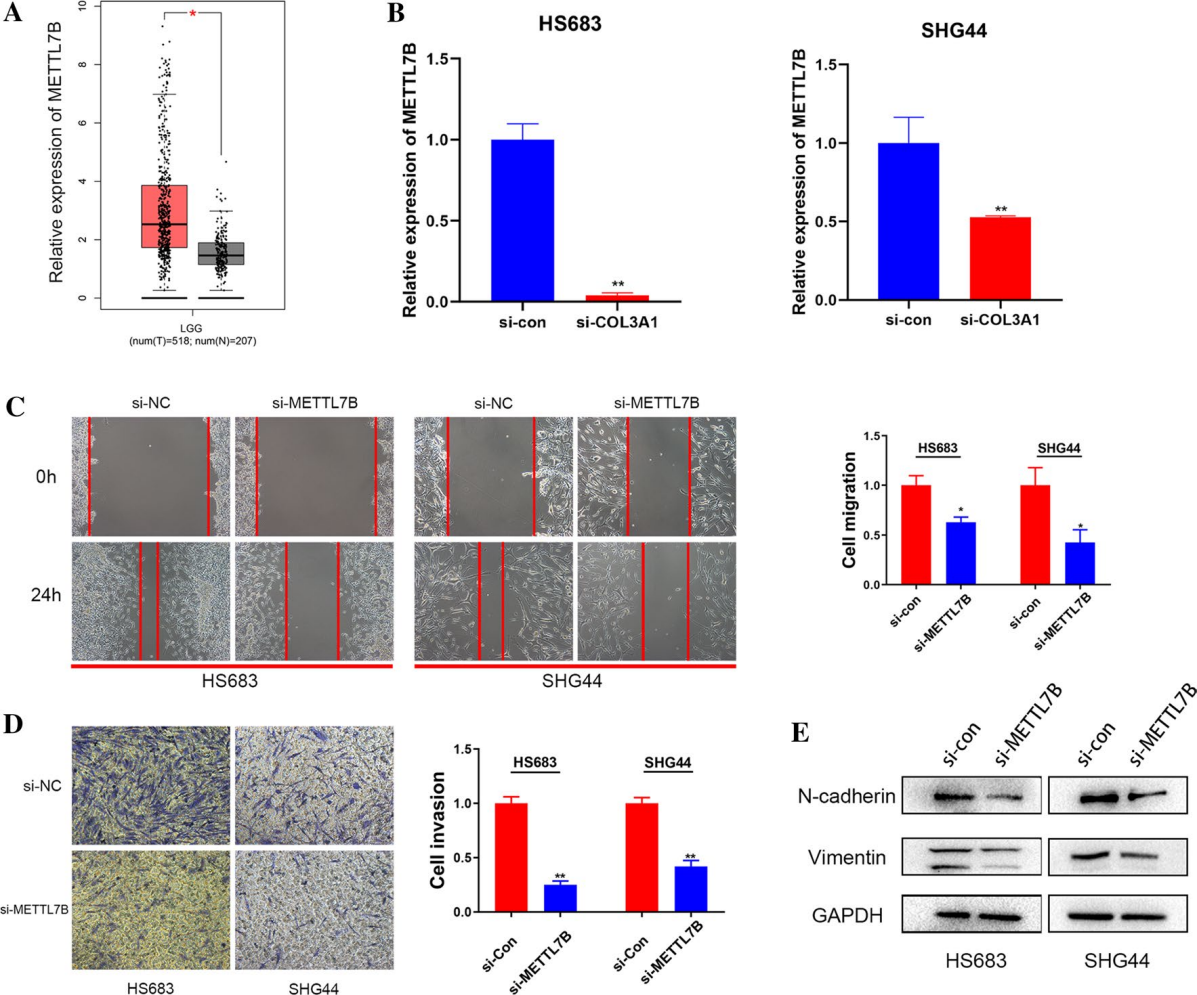
Knockdown of METTL7B inhibits migration, invasion, and EMT. **A** Relative expression of METTL7B in LGG and normal brain tissues. **B** The transfection efficiency of the METTL7B siRNA in HS683 and SHG44 cells, as measured using qRT-PCR. **C**–**D** Wound healing assays and Transwell assays indicated the migratory and invasive abilities of glioma cells after transfection. **E** Western blots showing levels of the Vimentin and N-cadherin proteins in HS683 and SHG44 cells after METTL7B knockdown with an siRNA. Data are represented as the mean values ± SD. * *p* < 0.05, and ** *p* < 0.01

We used LGG for the subsequent analysis to further validate whether the METTL7B gene was involved in tumor progression. Based on the GEPIA database, METTL7B was expressed at high levels in LGG samples compared with normal samples (Fig. 9A). We used a small interfering RNA (siRNA) to knockdown the METTL7B gene in HS683 and SHG44 cells and to explore the biofunction of METTL7B gene in LGG progress. METTL7B expression was substantially downregulated in HS683 and SHG44 cells transfected with siMETTL7B (Fig. 9B). We subsequently conducted scratch wound healing and Transwell assays to further confirm the migration and invasion of glioma cells after transfection with the siRNA. Wound healing and invasive activities were decreased after METTL7B knockdown (Fig. 9C, D). Because E-cadherin is expressed at very low levels in LGG cells, the levels of two common EMT markers (N-cadherin and Vimentin) were detected to analyze the EMT process in glioma. The levels of the N-cadherin and Vimentin proteins were significantly decreased after METTL7B knockdown in HS683 and SHG44 cells (Fig. 9E). Therefore, METTL7B contributes to the migration, invasion, and EMT process in LGG.

### METTL7B increases LGG cell proliferation and colony formation

**Fig. 10:**
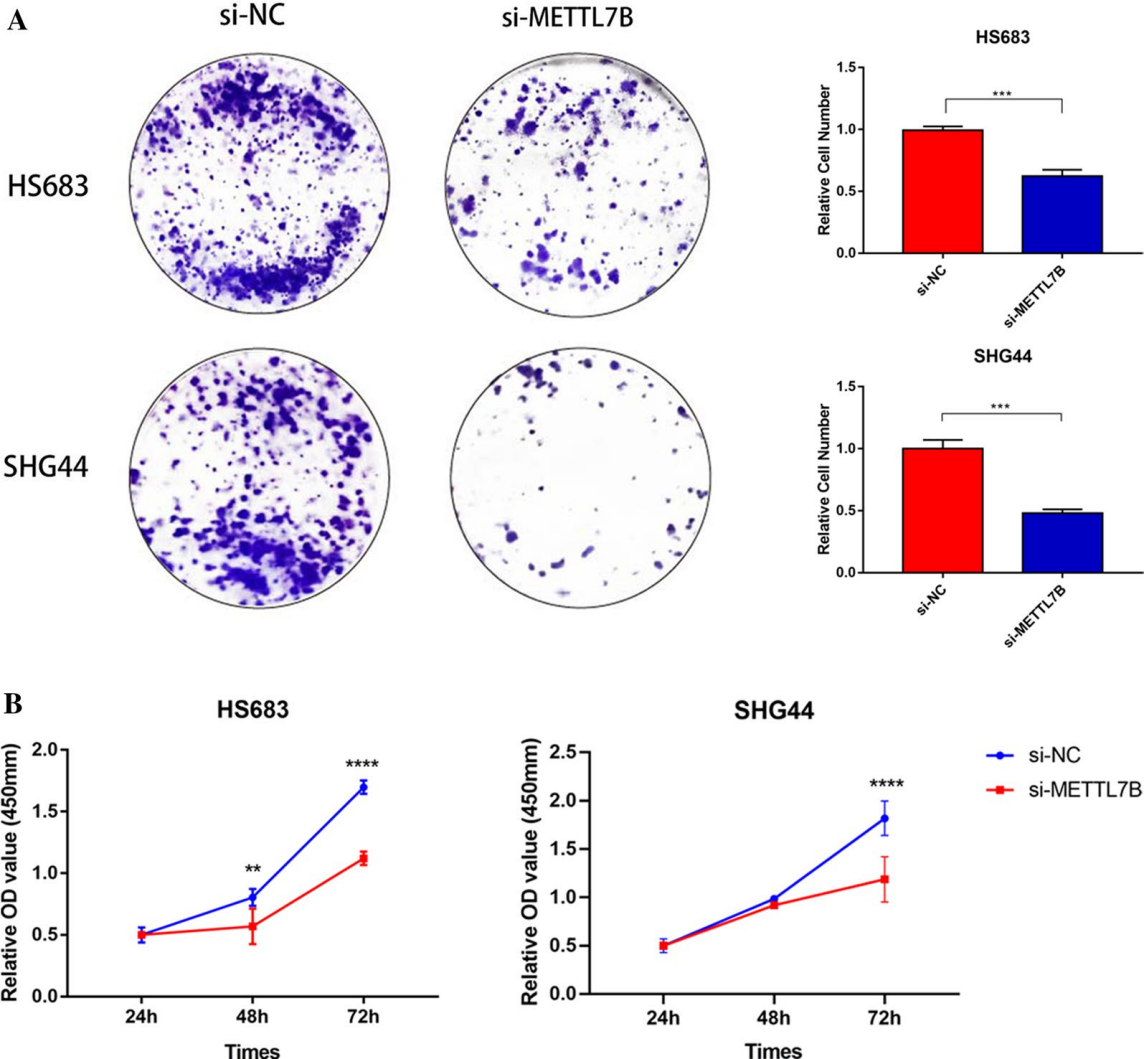
METTL7B alters the proliferation of LGG cells. **A** Colony formation experiments using HS683 and SHG44 showed reduced cell viability in the si-METTL7B group. **B** CCK-8 assays showed the suppressed proliferation ability of transfected LGG cells

Based on the aforementioned findings, we also conducted experiments on cell proliferation and colony formation in LGG. In a colony formation assay, knockdown of METTL7B in HS683 and SHG44 cells significantly decreased the number and volume of colonies (Fig. 10A). The CCK-8 assay was utilized to evaluate the effect of changes in the expression of the METTL7B gene on cell proliferation. Compared with the negative control (NC) group, the proliferation of HS683 and SHG44 cells was suppressed in the si-METTL7B group (Fig. 10B). Thus, the METTL7B gene affects cell proliferation, further confirming its carcinogenic activity.

## Discussion

METTL7B, a member of the methyltransferase-like protein (METTL) family, was reported to be involved in tumorigenesis and cancer progression [4–6, 8, 20]. However, its role in most cancers remains unclear. A pan-cancer analysis can determine the difference between tumor and normal tissues, providing a broader understanding of the mechanisms of cancer development and progression [21]. To date, many studies have investigated the association of a variety of genes and the prognosis of various cancers by performing a pan-cancer analysis. In this study, we performed a pan-cancer analysis of the expression, prognosis, and mutation of METTL7B in different types of tumor and normal tissues. METTL7B expression was increased in tumor tissues. In addition, METTL7B expression was positively correlated with the prognosis, immune cell infiltration, and immune checkpoint molecule expression. In addition, TMB and MSI analysis revealed their relationship with the expression of the METTL7B gene in different tumor types. Finally, cell-based experiments showed that METTL7B promotes the proliferation, migration, invasion, and EMT of LGG cells, which reflects the biological function of METTL7B in tumor progression.

Compared with normal tissues, METTL7B was reported to be expressed at high levels in ESCA [7], LUAD [5], and THCA [4]. By analyzing 27 cancer datasets, we found that METTL7B expression was upregulated in 22 tumors, including ESCA, LUAD and THCA, consistent with previous reports. In addition, our results revealed significant differences in METTL7B expression in some new tumor types, such as LGG. By analyzing different online databases, the results of the MEETL7B expression across cancers were often different. Therefore, our results must be further confirmed in various cancers. Our study suggests that METTL7B is abnormally expressed in a variety of cancers, providing insights into the application of prognostic markers for multiple cancer.

OS and DFS are important indicators of the disease prognosis in a survival analysis. By performing IHC staining of tissue specimens, Ali et al. found that patients with upregulated METTL7B expression had a reduced OS rate. Since METTL7B is also secreted into the extracellular space, the authors detected METTL7B levels in the serum of healthy controls and patients with LUAD, which represents an important detection method for clinical diagnosis and treatment [5]. By analyzing the OS and DFS of patients from 27 cancer datasets, we found significant differences in the expression of METL7B in various cancers. In particular, in LGG, the expression of METTL7B can be used as an important marker of a poor prognosis. Therefore, our studies provide a new insight into the prognosis and treatment of tumors, and we believe future studies focusing on the level of METTL7B in peripheral circulation may provide an important foundation for developing potential diagnostic biomarkers of cancer.

The TME and immune-related biomarkers play essential roles in the precise treatment of some cancers [22]. The TME, including immune cells, stromal cells, and cytokines, has been proven to determine the biological behavior of cancer cells [23–25]. Infiltrating immune and stromal cells account for the majority of normal cells in solid tumor tissue. Using a special approach, stromal and immune scores can be used to calculate the proportion of infiltrated stromal and immune cells in the tumor tissue. Tumor purity, which refers to the proportion of specific cancer cells in a tumor sample, is strongly correlated with gene expression, tumor biology, and clinical features [26, 27]. It can be inferred by analyzing the ESTIMATE score [28]. Lucas et al. observed a reduced T cell abundance in glioma with mutant IDH1 [29]. By analyzing the infiltration of immune cells and the stromal score, NFE2L2 gene was found to play an important role in tumor immunity [30]. Huadi et al. reported that the expression of CKMT1B is significantly correlated with the levels of infiltrating mast cells and M2 macrophages in LGG [31]. Fan et al. postulated that the immunoserotyping of diffuse LGG might play a critical role in clinical diagnosis and treatment [32]. An increasing number of studies have reported the immunomodulatory role of key genes in cancer. Similarly, our results implied that the expression of METTL7B was related to immune cell infiltration in LGG. Moreover, the relationship between the METTL7B expression and immune checkpoint markers shows the vital role of METTL7B in immunomodulation of the TME.

Immune checkpoint inhibitors (ICIs), which represent a new method for developing immunotherapies, have become a potential weapon in fighting different cancers [33]. The TMB and MSI are predictive biomarkers in patients with tumors receiving immunotherapy [34–36]. Patients with a high TMB or MSI are more likely to experience a long-term survival benefit from immunotherapy [37, 38]. To date, no similar studies of the TMB and MSI have been conducted on the METTL7B gene. Based on our results, METTL7B expression is associated with the TMB and MSI in different tumors. Several PD-1/PD-L1 ICIs have been approved to treat different cancers [39]. The prospect of immunotherapy for malignant tumors is developing rapidly, and an increasing number of ICIs are being developed to treat cancers. Thus, more research is needed to investigate the potential association between the METTL7B gene and ICIs, TMB and MSI.

METTL7B was initially considered one of the Golgi body-related methyltransferases [40]. In the advanced stage of thyroid cancer, METTL7B plays an important role in regulating the epithelial–mesenchymal transformation (EMT) induced by TGF-beta [5]. Liu et al. found that silencing METTL7B reduces cell proliferation by causing arrest in G0/G1 phase in cancer cells [8]. Functional annotation was performed in our studies to explore the potential mechanisms of METTL7B in LGG. The EMT was also involved in the development of LGG in our study. Moreover, METTL7B might participate in immune-related pathways and biological behaviors of LGG. Based on these bioinformatics analyses, we conducted in vitro experiments to investigate the biological functions of METTL7B in LGG cell lines. Consistent with previous findings, the METTL7B gene promoted the proliferation, migration and EMT process of LGG cells.

Although we conducted a pan-cancer analysis of many aspects of METTL7B using multiple databases, our study still has much room for improvement. First, the microarray and sequencing data were derived from the analysis of tumor tissue information, and thus the analysis of immune cell markers may be systematically biased at the cellular level. Therefore, more detailed studies, such as single-cell RNA sequencing, are needed. Second, limited experiments were conducted in this study, and we should perform more experiments in vivo or in vitro, especially in LGG. Third, the expression of METTL7B was related to tumor immunity and the disease prognosis. We cannot yet infer whether METTL7B affects survival outcomes by exerting immunological effects. Future studies of the METTL7B gene may address these issues.

## Conclusions

We conducted a systematically pan-cancer analysis and a series of in vitro experiments to learn more about the METTL7B gene. Consistent with the results previous studies, METTL7B is aberrantly overexpressed in most cancer types and represents a potential prognostic predictor for many tumors. Moreover, the METTL7B gene plays a vital role in regulating the immunity of the TME, especially in LGG. By performing a Gene Set Enrichment Analysis, we identified associations of the METTL7B gene with multiple pathways. In vitro experiments further confirmed that METTL7B is involved in the development and progression of LGG. Therefore, METTL7B might be a novel marker gene for predicting the prognosis of and a potential therapeutic target in LGG. However, our studies still have some limitations. Further experiments on the functional mechanism and even clinical trials are warranted to validate our conclusions and have a better understanding of the METTL7B gene.

## Data Availability

The datasets used in the current study are included within the article. Other data and materials are available from the corresponding author upon reasonable request.

## Abbreviations

ACC: Adrenocortical carcinoma
BLCA: Bladder urothelial carcinoma
BRCA: Breast invasive carcinoma
CESC: Cervical squamous cell carcinoma
CHOL: Cholangiocarcinoma
COAD: Colon adenocarcinoma
DLBC: Lymphoid neoplasm diffuse large B cell lymphoma
ESCA: Esophageal carcinoma
GBM: Glioblastoma multiforme
LGG: Brain lower grade glioma
HNSC: Head and neck squamous cell carcinoma
KICH: Kidney chromophobe
KIRC: Kidney renal clear cell carcinoma
KIRP: Kidney renal papillary cell carcinoma
LAML: Acute myeloid leukemia
LIHC: Liver hepatocellular carcinoma
LUAD: Lung adenocarcinoma
LUSC: Lung squamous cell carcinoma
MESO: Mesothelioma
OV: Ovarian serous cystadenocarcinoma
PAAD: Pancreatic adenocarcinoma
PCPG: Pheochromocytoma and paraganglioma
PRAD: Prostate adenocarcinoma
READ: Rectum adenocarcinoma
SARC: Sarcoma
SKCM: Skin cutaneous melanoma
STAD: Stomach adenocarcinoma
TGCT: Testicular germ cell tumors
THCA: Thyroid carcinoma
THYM: Thymomas
UCEC: Uterine corpus endometrial carcinoma
UCS: Uterine carcinosarcoma
UVM: Uveal melanoma
